# Long-Term Outcomes of Coronary Artery Aneurysms in Children With Kawasaki Disease: A Systematic Review

**DOI:** 10.7759/cureus.94418

**Published:** 2025-10-12

**Authors:** Shoaib Syed Mohammed Shafy, Karla Andrea Calderón Salavarria, Sara Saleh, Ingrid Cuino, Sadaf Nadeem, Rebeca Cristina Romero Perez, Arushi Shetty, Kedar Adhikari, Tanya Khatter, Chinmayee Arasada, Naji Moussa, Eziamaka Mbaekwe, Panchanit Horsaengchai, Ramsha Ali

**Affiliations:** 1 Critical Care Medicine, Yenepoya Medical College and Research Center, Mangalore, IND; 2 Medicine, Catholic University Santiago of Guayaquil, Guayaquil, ECU; 3 Medicine, Hashemite University, Zarqa, JOR; 4 Acute Medical Unit, Shrewsbury and Telford Hospital, Shrewsbury, GBR; 5 Medicine, Dow University of Health Sciences, Karachi, PAK; 6 Medicine, La Universidad del Zulia, Maracaibo, VEN; 7 Medicine, Pravara Institute Of Medical Sciences, Ahmednagar, IND; 8 Medicine, University of Science and Technology, Chittagong, BGD; 9 Medicine, School of Medical Science and Research, Sharda University, Greater Noida, IND; 10 Internal Medicine, M.S. Ramaiah Medical College, Bangalore, IND; 11 Internal Medicine, Richmond Gabriel University, Saint Vincent, VCT; 12 Oncology, St. George’s University Hospital, London , GBR; 13 Medicine, Royal College of Surgeons in Ireland (RCSI) and University College Dublin (UCD) Malaysia Campus, George Town, MYS; 14 Medicine and Surgery, Peoples University of Medical and Health Sciences, Hyderabad, PAK

**Keywords:** cabg, coronary artery aneurysm, ivig, kawasaki disease, mace, pci, pediatric vasculitis

## Abstract

Kawasaki disease (KD) is a systemic inflammation of the blood vessels seen in children. It is the leading cause of acquired heart disease. Coronary artery aneurysm (CAA) is the most concerning complication, associated with long-term morbidity and mortality. This systematic review examined long-term outcomes of CAAs in KD, with emphasis on regression, complications, and the role of medical and surgical interventions. A total of 21 studies involving 10,922 patients were reviewed. The incidence of CAAs ranged from 3%-27%, mostly affecting infants under 6 months. Small and moderate aneurysms regressed in the majority of cases (>80%), whereas giant aneurysms were less likely to regress. Complications included thrombosis, myocardial infarction, major adverse cardiac events, and cardiac death. Early treatment with intravenous immunoglobulin (IVIG) enhanced the likelihood of regression. Aneurysms in the left anterior descending artery were less likely to regress, suggesting an anatomical influence. Coronary artery bypass grafting was the most common surgical intervention. Early and aggressive management with IVIG, with escalation to steroids or biologics when required, remains the preferred approach. Surgical intervention is sought for giant or persistent aneurysms. These findings emphasize the importance of timely diagnosis, risk stratification, and the establishment of standardized guidelines to optimize long-term care and surveillance of patients with KD.

## Introduction and background

Kawasaki disease (KD) is a pathology that represents a diagnostic challenge in pediatrics. Although therapeutic strategies have improved over the years, this condition remains the leading cause of acquired cardiovascular disease in children, with an increased prevalence of 218.6 per 100,000 children. This raises an important question: How can we better predict and prevent these long-term outcomes? [1].

This syndrome is a vasculitis of unknown etiology affecting medium-caliber arteries in children under five. Two types have been described in the medical literature: the typical form, in which the patient meets all the diagnostic criteria (five-day fever, non-purulent conjunctival injection, generalized rash, changes in palms and soles, cervical lymphadenopathy, and erythema of the lips), and the incomplete form, which poses a major diagnostic challenge [2]. Among the complications of the disease, cardiac involvement is the most significant, constituting the main cause of long-term morbidity and mortality. These serious problems are characterized by having their peak between the acute and subacute periods of the disease, so their timely and early detection is of utmost importance. They can include non-specific alterations in the electrocardiogram, myocardial inflammation, coronary aneurysms, and sudden death [3].

The formation of aneurysms is the most relevant complication within cardiovascular problems, and without correct treatment, more than 25% of patients with this disease can develop it [4]. Dilation can range from small to large or giant aneurysms, which may persist for years and carry a significant risk of thrombosis and stenosis. In some cases, this can progress and interact with risk factors for atherosclerosis in adulthood, potentially leading to ischemia and infarction. It is estimated that approximately 5% of heart attacks that occur in adulthood are the result of the sequelae of KD suffered during childhood [5]. Every patient with coronary aneurysms has a lifelong risk of thrombosis, stenosis, and acute coronary syndromes. To prevent long-term fatal outcomes, close monitoring and early identification of high-risk patients are essential; therefore, echocardiography remains the primary diagnostic tool.

The severity of this disease is directly related to the residual cardiac pathology (coronary artery involvement), which makes frequent follow-up extremely important [6]. The long-term outcomes of the cardiac complications are not yet fully understood, and these events can occur years or even decades after initial symptoms [1,7]. Multiple studies have investigated treatments such as glucocorticoids, intravenous immunoglobulin (IVIG), and aspirin. However, optimal dosing, combination strategies, and the overall efficacy of these therapies in reducing CALs remain subjects of debate [2,8]. Given the potential of these serious outcomes, a thorough understanding of the natural history of coronary artery aneurysm (CAA) is critical to improve and preserve cardiac health and decrease morbidity in patients with KD.

The increasing recognition of these long-term complications associated with this pathology emphasizes the urgent need for an extensive review of the existing literature and evidence to guide clinical practice among pediatricians. Based on variations in treatment response and follow-ups, it is evident that the existence of persistent gaps in care leaves some children vulnerable to preventable complications [8]. By addressing these questions and concerns, this study may contribute to reducing long-term cardiovascular complications in pediatric patients with KD, consolidating current knowledge on CAAs associated with this illness and their implications, and providing clinicians and physicians with evidence-based insights to improve monitoring, intervention, and patient outcomes.

This systematic review compiles findings from multiple studies addressing the long-term cardiovascular complications and their clinical implications. Based on limited retrospective data, our evidence collection suggests a possible association between prior heart damage and an increased risk of ischemic events and myocardial infarction in adulthood. The findings underscore the critical role of early diagnosis, long-term follow-up, and proactive cardiovascular monitoring to mitigate future adverse outcomes. Exercise stress echocardiography offers a more useful and non-invasive method for detecting inducible myocardial ischemia in high-risk patients [3]. In addition, echocardiography allows for the measurement of the *Z*-score of the internal diameter of coronary aneurysms. Various studies have shown that while small to medium aneurysms with low *Z*-scores are more likely to regress, patients with giant CAAs or higher *Z*-scores at diagnosis are at greater risk of developing major cardiovascular events over time. This reinforces the usefulness of this tool both in risk stratification and in clinical follow-up [9].

There have been correlations between flow-mediated dilation (FMD) and the severity of CALs when we assess endothelial dysfunction in these patients [4]. We found biomarkers, such as NT-proBNP and neutrophil-to-lymphocyte ratio (NLR), that can be helpful in the prediction and prevention of CAAs [5,7,10]. Other markers, such as C-reactive protein, which is easily accessible in the hospital setting, have been studied together with serum amyloid-A, proving to be reliable markers to predict myocardial damage, regardless of the pre-existence of cardiac alterations in pediatric patients [11]. Moreover, further research has been conducted on certain parameters, such as the A/G ratio, revealing that cutoff points lower than 1.32 are directly related to an increased risk of developing coronary aneurysms [12].

It has been confirmed that the use of aspirin plus IVIG as standard therapy, compared to monotherapy with aspirin, significantly reduces the risk of CALs in non-IVIG-resistant patients. Therefore, combining IVIG with other drugs, such as prednisolone, has demonstrated significant advantages in preventing coronary abnormalities, reducing the need for additional interventions, and rapidly decreasing the inflammatory response in patients with KD [13,14]. In contrast, the efficacy of alternative treatments, such as anti-TNFα agents (e.g., infliximab and etanercept) and clarithromycin, in reducing the risk of CALs remains controversial in cases of IVIG-resistant disease [1,10]. Nevertheless, some studies, including the RAISE trial, suggest that incorporating glucocorticoids into initial management may help reduce inflammatory markers (e.g., erythrocyte sedimentation rate), fever duration, and hospitalization stay. However, these outcomes are not universally observed and may vary across different populations [13,15].

This review focuses on pediatric patients diagnosed with KD, particularly those who developed CAAs, and compares them with healthy children to investigate long-term cardiovascular outcomes. The review excludes adult patients and studies that concentrated on non-cardiovascular outcomes or had follow-ups shorter than one year. The studies used different diagnostic methods, imaging techniques, and treatment strategies to assess these cardiovascular events.

This study aims to analyze the long-term cardiovascular outcomes associated with CAAs in patients with a history of KD. By analyzing these results, this systematic review of the literature seeks to encourage the improvement of current clinical practice guidelines to prevent long-term cardiovascular complications in the pediatric population.

This article was previously presented as a Poster at the “Third World Conference on Pediatrics, Neonatology and Infectious Diseases held on July 14-15 in 2025, in London, United Kingdom.

Material and methods

This systematic review has been reported in concordance with the guidelines provided by Preferred Items for Systematic Review and Meta-Analysis (PRISMA) (Appendix) [16].

Data Sources and Search Strategy

The literature search was conducted systematically on PubMed in April 2025 with MeSH terms: ("Mucocutaneous Lymph Node Syndrome" [MeSH Terms] OR ("mucocutaneous" AND "lymph" AND "node" AND "syndrome") OR "Mucocutaneous Lymph Node Syndrome" OR ("kawasaki" AND "disease") OR "kawasaki disease" AND ("Coronary Aneurysm" [MeSH Terms] OR ("coronary" AND "aneurysm") OR "Coronary Aneurysm" OR ("coronary" AND "artery" AND "aneurysms") OR "coronary artery aneurysms")) OR "Coronary Aneurysm" [MeSH Terms] OR "Mucocutaneous Lymph Node Syndrome" [MeSH Terms].

Used in various combinations. After database search and removal of duplicates, the shortlisted studies were screened by four reviewers using title and abstract; only studies in the English language were selected, and those performed on human specimens. Exclusion criteria were implemented on the remaining articles, discarding studies after full-text screening based on irrelevance, poor quality, and ineligible outcomes.

Inclusion and Exclusion Criteria

The criteria for inclusion and exclusion of studies were fixed after discussion with the authors. The inclusion criteria for this review were clinical trials (randomized or non-randomized) or observational studies (prospective or retrospective cohorts and case-control studies) of pediatric patients (< 18 years of age) with confirmed KD who have CAAs with at least one long-term cardiovascular outcome (follow-up ≥1 year). Only studies published in English were included, with no restriction on publication date.

Studies were excluded if they had a follow-up period of less than one year, were case reports, reviews, or editorials. Studies were excluded if they involved animals or in vitro models, or lacked specific data on coronary artery anomalies (CAAs) or cardiovascular outcomes.

Data Extraction and Risk of Bias Assessment

The authors performed data extraction on characteristics of each study and its patient population, including study type, year of publication, sample size, population, any interventions or exposure, control group, primary outcome (coronary vascular disease), and secondary outcomes that met the inclusion criteria.

The quality of each study was interpreted by measuring the risk of bias for randomized controlled trials (RCTs) using the Cochrane Collaboration Risk of Bias tool [17]. The performance bias, selection bias, reporting bias, detection bias, attrition bias, and other biases were estimated for each RCT. We scored the risk of bias in each category as low, high, or unclear. In many of the included RCTs, the probability of performance bias was high as blinding of the surgeons, investigators, and patients was not executed. Some trials showed a low risk of selection bias, whereas all the RCTs showed a low risk of attrition and reporting bias. As far as the observational cohort studies are concerned, the risk of bias was assessed using the Critical Appraisal Skills Program (CASP) checklist [18]. The CASP checklist was utilized because it included clear and explicit guidance on how to respond to the items in the CASP checklist for each study. It could also be applied to the studies included in our systematic review, with minimal adaptation required to the CASP checklist. The CASP review checklist has three parts: Part A, Are the results of the study valid? Part B: What are the results? Part C: Will the results help locally (in the case of this review, interpreted as improving long-term cardiovascular outcomes in KD)? For each item, a response of "yes" (2), "no" (0), or "can’t tell" (1) was given. The studies were independently appraised by two reviewers, with any disagreements resolved via discussion. Results of the CASP checklist are provided in the Results Section (Tables 1-3) [19,20].

**Table 1:** CASP checklist for cohort studies. CASP, Critical Appraisal Skills Program

Questions	Akagi et al., 1992	Friedman et al., 2016	Miura et al., 2018	McCrindle et al., 2020	Kato et al., 2023	Koyama et al., 2022	Kato et al., 1982	Tsuda et al., 2017	Cameron et al., 2018	Liu et al., 2022	Salgado et al., 2017	Tang et al., 2018	Ogata et al., 2013	Santimahakullert et al., 2022	Chidambaram et al., 2023
Did the study address a clearly focused issue?	Y	Y	Y	Y	Y	Y	Y	Y	Y	Y	Y	Y	Y	Y	Y
Was the cohort recruited in an acceptable way?	Y	Y	Y	Y	Y	Y	?	?	Y	?	Y	Y	Y	Y	Y
Was the exposure accurately measured to minimize bias?	Y	Y	Y	Y	?	Y	?	Y	Y	?	Y	Y	?	?	Y
Was the outcome accurately measured to minimize bias?	Y	Y	Y	?	Y	Y	?	Y	?	Y	?	?	N	Y	?
Have the authors identified all important confounding factors?	?	N	Y	N	N	N	N	?	?	?	N	N	?	N	Y
Have the authors taken account of the confounding factors in design and/or analysis?	?	N	Y	N	N	N	?	?	?	?	N	N	Y	?	Y
Was the follow-up of subjects complete enough?	Y	Y	Y	Y	Y	Y	Y	Y	Y	Y	Y	Y	Y	Y	Y
Was the follow-up of subjects long enough?	?	Y	Y	Y	Y	Y	Y	?	?	Y	N	?	Y	?	Y
What are the results of this study?	Y	Y	Y	Y	Y	Y	Y	Y	Y	Y	Y	Y	Y	Y	Y
How precise are the results?	Y	Y	Y	Y	Y	Y	Y	?	Y	?	Y	Y	Y	Y	Y
Do you believe the results?	Y	Y	Y	Y	Y	Y	Y	Y	Y	Y	Y	Y	Y	Y	Y
Can the results be applied to the local population?	?	?	?	?	N	Y	?	?	?	N	?	?	N	?	?
Do the results of this study fit with other available evidence?	Y	Y	Y	Y	Y	Y	Y	Y	Y	Y	Y	Y	?	Y	Y
What are the implications of this study for practice?	Y	Y	Y	Y	Y	Y	Y	Y	Y	?	Y	Y	?	Y	Y
Score out of 28	24	23	27	22	21	24	21	22	23	20	20	21	20	22	26

**Table 2:** CASP checklist for case-control studies. CASP, Critical Appraisal Skills Program

Questions	Liu et al., 2012	Shah et al., 2015
Did the study address a clearly focused issue?	Y	Y
Did the authors use an appropriate method to answer their question?	N	?
Were the cases recruited in an acceptable way?	?	Y
Were the controls selected in an acceptable way?	Y	Y
Was the exposure accurately measured to minimize bias?	Y	Y
Aside from the exposure, did the groups have similar characteristics?	Y	Y
Have the authors taken account of the potential confounding factors in the design and/or in their analysis?	Y	Y
Was the treatment effect large?	?	?
Was the estimate of the treatment effect precise?	?	?
Do you believe the results?	Y	Y
Can the results be applied to your patients/population of interest?	Y	Y
Do the results of this study fit with other available evidence?	Y	Y
Score out of 24	19	21

**Table 3:** CASP checklist for cross-sectional studies.

Questions	Mitani et al., 2005
Did the study address a clearly focused issue?	Y
Did the authors use an appropriate method to answer their question?	N
Were the subjects recruited in an acceptable way?	?
Were the measures accurately measured to reduce bias?	Y
Were the data collected in a way that addressed the research issue?	Y
Did the study have enough participants to minimize the chance of play?	Y
How are the results presented, and what is the main result?	Y
Was the data analysis sufficiently rigorous?	Y
Is there a clear statement of findings?	Y
Can the results be applied to the local population?	N
How valuable is the research?	Y
Score out of 22	17

## Review

Results

Literature Review and Study Characteristics

**Figure 1:**
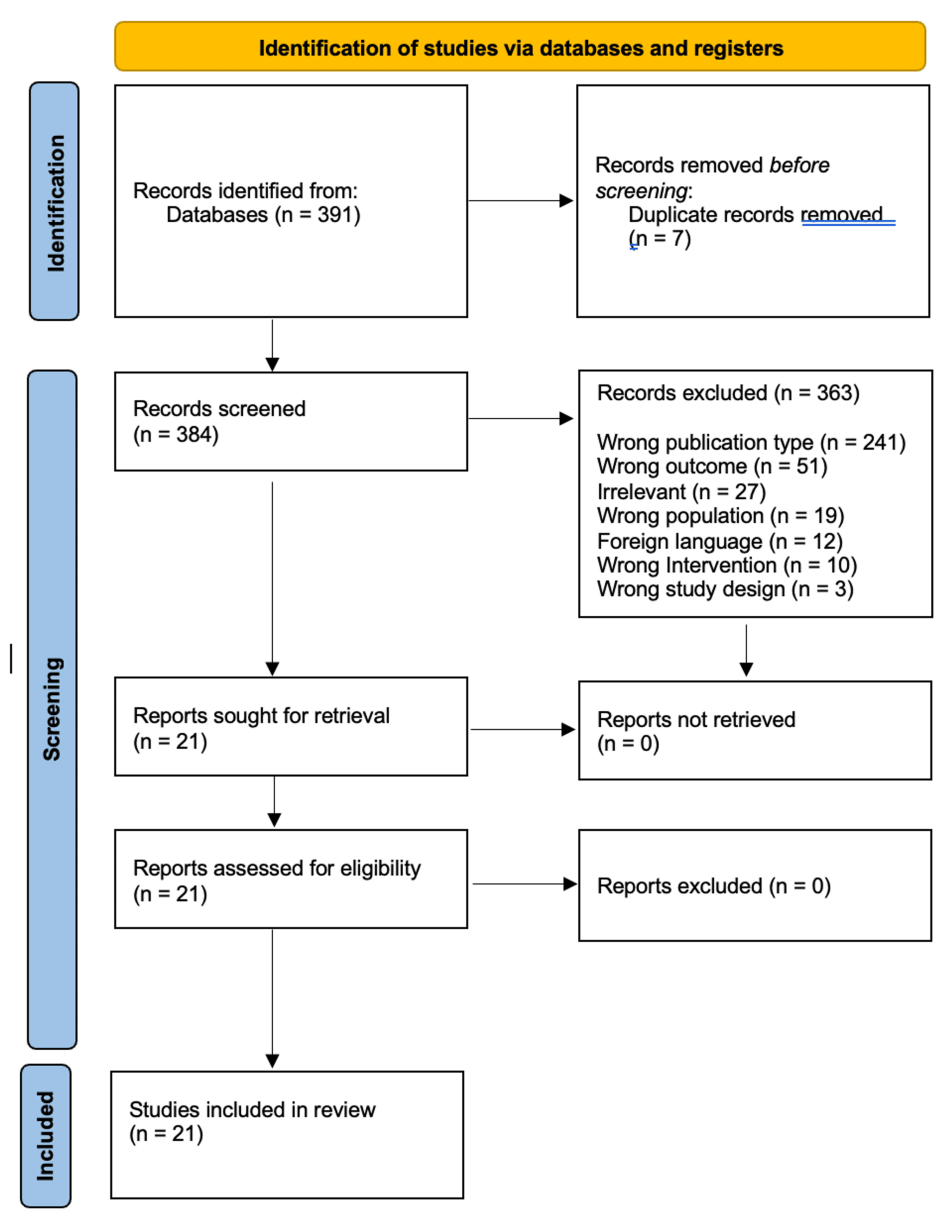
PRISMA 2020 flow diagram for systematic reviews, including searches of electronic databases. We searched PubMed along with manual reference screening. After removing 7 duplicates, 384 records were screened, of which 363 were excluded for reasons such as ineligible population, outcomes not related to coronary artery aneurysms, or insufficient data. A total of 21 studies were included in the systematic review. PRISMA, Preferred Reporting Items for Systematic Reviews and Meta-Analyses Adapted from the PRISMA 2020 statement (CC BY 4.0). To view a copy of this license, visit https://creativecommons.org/licenses/by/4.0/

The PRISMA flowchart summarizes the search and study selection process (Figure 1). In our initial search, we identified 391 studies from PubMed using the keywords "Kawasaki disease", "Mucocutaneous lymph node syndrome", "Coronary Aneurysm and Pediatric vasculitis". After removing duplicates, 384 studies remained, and we excluded 583 studies based on titles and abstracts. We accessed the full text of the remaining 253 studies. We excluded 173 based on the exclusion criteria. Ultimately, we included 21 studies. Of all the included articles, 3 were randomized control trials, 15 were cohort studies, 2 were case control, and 1 was a cross-sectional study; all 21 articles were from the United States, Canada, the United Kingdom, Japan, India, China, and Taiwan. Study characteristics and baseline characteristics of participants are provided in Table 4. 

**Table 4:** Study characteristics and key outcomes of included studies in this review. RCT, randomized controlled trials; CAA, coronary artery aneurysm; NA, not applicable; IVIG, intravenous immunoglobulin; MI, myocardial infarction; CABG, coronary artery bypass grafting; MACE, major adverse cardiovascular events; CE, coronary events; CA, coronary artery; A/G, albumin/globulin; MPV, mean platelet volume; PDW, platelet distribution width; NLR, neutrophil-to-lymphocyte ratio; CAL, coronary artery lesion; JAHA, Journal of the American Heart Association; AHA, American Heart Association; BMJ, British Medical Journal

Study	Design	Country	Sample size	Male, *n* (%)	CAA, *n* (%)	Primary outcome	References
Miura et al., 2018	Cohort	Japan	1006	714 (71%)	NA	CE, MACE, coronary interventions	[9]
Mitani et al., 2005	Cross sectional	Japan	80	53 (66.25%)	-	Correlation between inflammatory markers and CAA	[11]
Liu et al., 2022	Cohort	China	484	345 (71.3%)	130 (26.9%)	A/G ratio, CA progression	[12]
Kobayashi et al., 2012	RCT	Japan	248	122 (49.2%)	32 (13%)	Incidence of CAA	[13]
Portman et al., 2019	RCT	United States	201	127 (63.2%)	44 (21.9%)	IVIG resistance	[14]
Liu et al., 2012	Case-Control	China	469	280 (59.7%)	108 (23%)	MPV, PDW	[15]
Akagi et al., 1992	Cohort	Canada	583	-	80 (13.7%)	MI, CABG, prognosis of aneurysms	[21]
Friedman et al., 2016	Cohort	United States	500	360 (72%)	-	CAA regression, MACE	[22]
McCrindle et al., 2020	Cohort	Canada, United States, Taiwan	1651	1184 (72%)	NA	Lumen narrowing, CA revascularization, CA thrombosis, MI, MACE	[23]
Kato et al., 2023	Cohort	Japan	1369	970 (70.9%)	-	CAA regression	[24]
Koyama et al., 2022	Cohort	Japan	179	137 (76.5%)	NA	Coronary events	[25]
Kato et al., 1982	Cohort	Japan	290	36 (83.7%)	43 (15%)	CAA prognosis	[26]
Tsuda et al., 2017	Cohort	Japan	579	-	127 (21.9%)	MI free rate, CA re-vascularization free rate	[27]
Cameron et al., 2018	Cohort	United States	250	147	81 (32.4%)	Mean *Z*-score	[28]
Salgado et al., 2017	Cohort	United States	720	447 (62.1%)	118 (16.4%)	CAA	[29]
Ogata et al., 2013	Cohort	United States, Japan	1082	659 (60.9%)	398 (36.8%)	Median *Z*-score	[30]
Hamada et al., 2019	RCT	Japan	173	99 (57.2%)	39 (22%)	Incidence of CAA	[31]
Tang et al., 2018	Cohort	China	666	-	120 (18%)	CAA regression	[32]
Shah et al., 2015	Case-Control	United Kingdom	143	-	38 (26.6%)	Myocardial function, arterial stiffness	[33]
Chidambaram et al., 2023	Cohort	India	79	56 (70.9%)	32 (40.5%)	NLR, CAL	[34]
Santimahakullert et al., 2022	Cohort	Thailand	170	110 (64.7%)	NA	MACE	[35]

Risk of Bias in Included Studies

**Figure 2:**
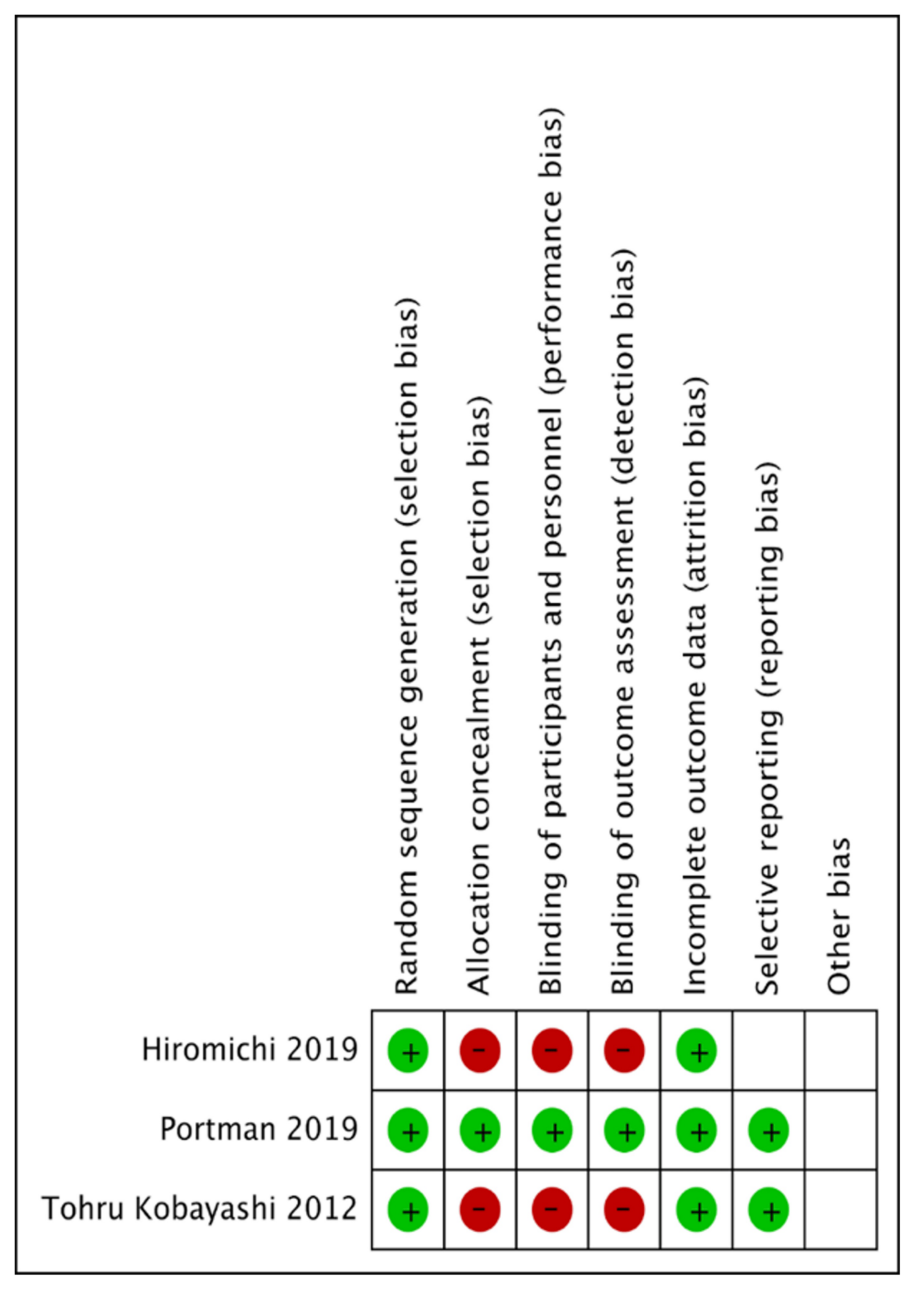
Risk of bias assessment of RCTs using the Cochrane Collaboration Risk of Bias tool. Cochrane Collaboration Risk of Bias tool [17]. RCT, randomized controlled trial

**Figure 3:**
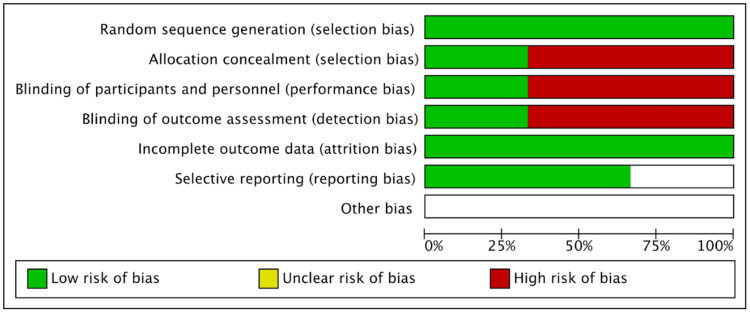
Risk of bias graph of RCTs using the Cochrane Collaboration Risk of Bias tool. Cochrane Collaboration Risk of Bias tool [17]. RCT, randomized controlled trial

We assessed the risk of bias of each included study based on the seven domains of the Cochrane Collaboration Risk of Bias tool (RoB 1). An overall summary of bias present within each of the included studies is presented in Figures 2-3. No studies were at low risk of bias in all domains. Of the included studies, two studies had at least one domain at high risk of bias. No study was at high risk of bias in all domains. All studies were at unclear risk of bias in at least one domain.

Random Sequence Generation

All trials included randomly assigned patients either to the intervention or control group and were deemed to be trials with a low risk of bias.

Allocation Concealment

Allocation concealment (selection bias) was reported in all trials. Of the three included trials, two trials did not adhere to the allocation of concealment and hence were classified as a high risk of bias [14,16]. On the other hand, one trial met the requirements for allocation concealment and hence was classified as a low risk of bias [31].

Blinding of Participants and Personnel

Blinding of participants and personnel was reported in 1 trial, and therefore, we classified it as low risk of bias [31]. In the two other trials, patients and physicians were not masked to assignment, and hence, these trials were classified as a high risk of bias [14,16].

Blinding of Outcome Data Assessment (Attrition Bias)

In one trial outcome, assessors were blind to participant group allocation; therefore, we classified them as low risk of bias [31]. In the two other trials, patients and physicians were not subjected to blinding of outcome assessment, and so were classified as having a high risk of bias [14,16].

Incomplete Outcome Data Reported

All included trials reported that there were no participant withdrawals or significant losses to follow-up affecting the analyses in any of the trials. One trial reported loss of echocardiographic data of one participant in the intervention group and was excluded from the trial [14]. Hence, all trials were considered low risk of bias for this domain [14,16,31].

Selective Reporting (Reporting Bias)

We classified two trials [16,31] as low risk of bias because protocols were available for each trial. One trial [14] was categorized as unclear risk of bias due to discrepancies in the definition of CAA as determined by Japanese and International criteria in the study.

Quality Assessment for Observational Studies

CASP scores for individual studies are presented in the Supplemental file. The reviews generally addressed whether the results of the review were valid and were judged to have included the relevant papers. However, in three of the studies [15,24,32], results could not be applied to the local population, and four of the reviews [24,29,30,32] could have carried out a more rigorous quality assessment. All of the reviews clearly presented the findings of the review, the findings were relevant to safety improvement in primary care, and all of the outcomes have been adequately considered.

Study Outcomes

Severity of CAAs and associated risk of adverse events: The size of the CAA is unequivocally the most critical determinant of prognosis, influencing both the likelihood of regression and the risk of MACE. Patients with large or giant CAAs (defined by a *Z*-score ≥10 or an absolute diameter ≥8 mm) face a substantially elevated risk of complications, including luminal narrowing, thrombosis, myocardial infarction (MI), and the need for revascularization [21-23].

**Figure 4:**
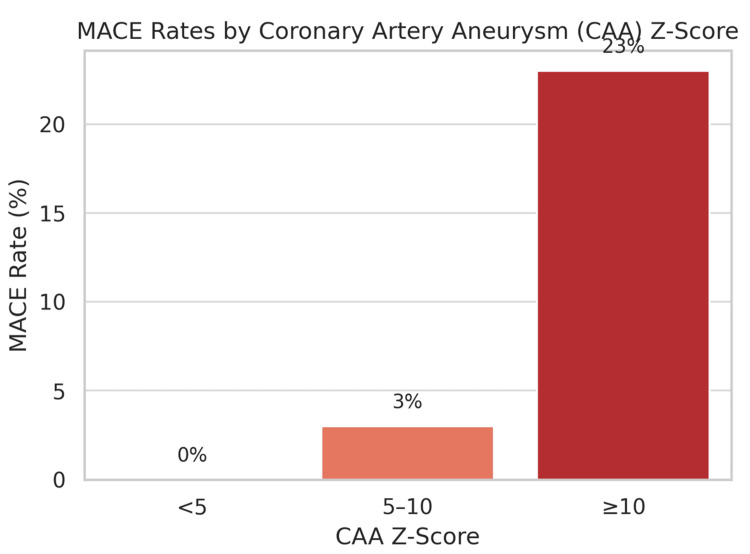
Patients with KD with higher Z-scores are more likely to develop MACE. Original figure created by the authors. KD, Kawasaki disease; MACE, major adverse cardiovascular event

Data showed that MACE occurred in 23% of patients with a *Z*-score ≥10 at diagnosis, compared to 3% for *Z*-scores between 5 and 10, and 0% for *Z*-scores <5 (Figure 4). This finding is mirrored across studies, with no MACE reported in patients with small CAAs.

An international registry further refined this risk, demonstrating that for branches with CAA *Z*-scores ≥10, the 10-year cumulative incidence of luminal narrowing was 20% ± 3%, coronary artery thrombosis was 18% ± 2 %, and composite MACE was 14% ± 2 %. A particularly high-risk subgroup is identified by CAA *Z*-scores ≥ 20, which carry a distinctly greater risk profile compared to those with *Z*-scores between 10 and 20. Male sex is consistently associated with a higher occurrence of coronary events (CE) and MACE.

Demographic and Clinical Predictors of Outcome

**Figure 5:**
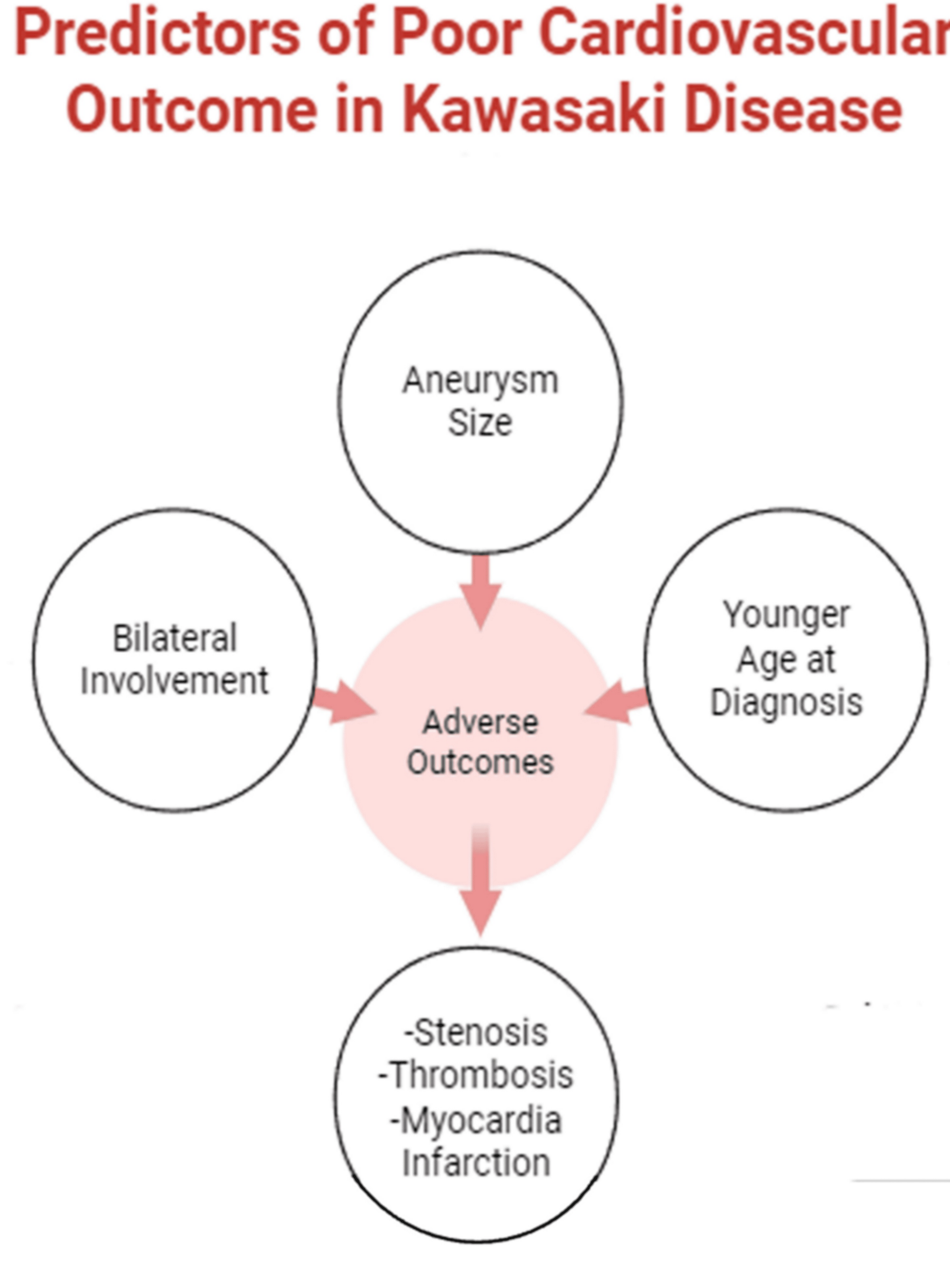
Aneurysm size, Age at diagnosis and Bilateral involvement are all found to be associated with worse adverse events including Stenosis, Thrombosis and Myocardial Infarction. Created with BioRender.com.

Age at diagnosis significantly impacts severity, with infants, particularly those under one year of age (and especially those <6 months), exhibiting a higher prevalence and severity of CAAs. One study noted a mean maximum *Z*-score of 3.37 in infants versus 2.07 in older children (*P* < 0.001), along with higher incidences of medium (11% vs. 3%, *P *= 0.015) and giant aneurysms (8% vs. <1%, *P *= 0.005) in infants. Even with timely treatment, nearly 20% of infants under six months developed aneurysms or giant aneurysms (Figure 5).

Delayed diagnosis, defined as treatment initiated after day 10 of illness, is consistently linked to an increased risk and severity of CAA formation. Patients who did not receive intravenous IVIG experienced a significantly higher incidence of MACE (25% vs. 1.4% in those treated within 10 days). The absence of timely IVIG treatment is an independent risk factor for MACE.

Biomarkers and CAA Outcomes

A lower A/G ratio is negatively correlated with the development of mid-to-large-sized CAAs. A cutoff of 1.32 for an A/G ratio had a sensitivity of 79% and specificity of 49% for predicting mid-to-large-sized CAA development. The neutrophil-to-lymphocyte ratio (NLR) ≥2.08 between days 4 and 6 of fever onset reliably predicted the development of coronary artery lesions (CALs), showing 82% sensitivity and 80% specificity.

CAA Regression

**Figure 6:**
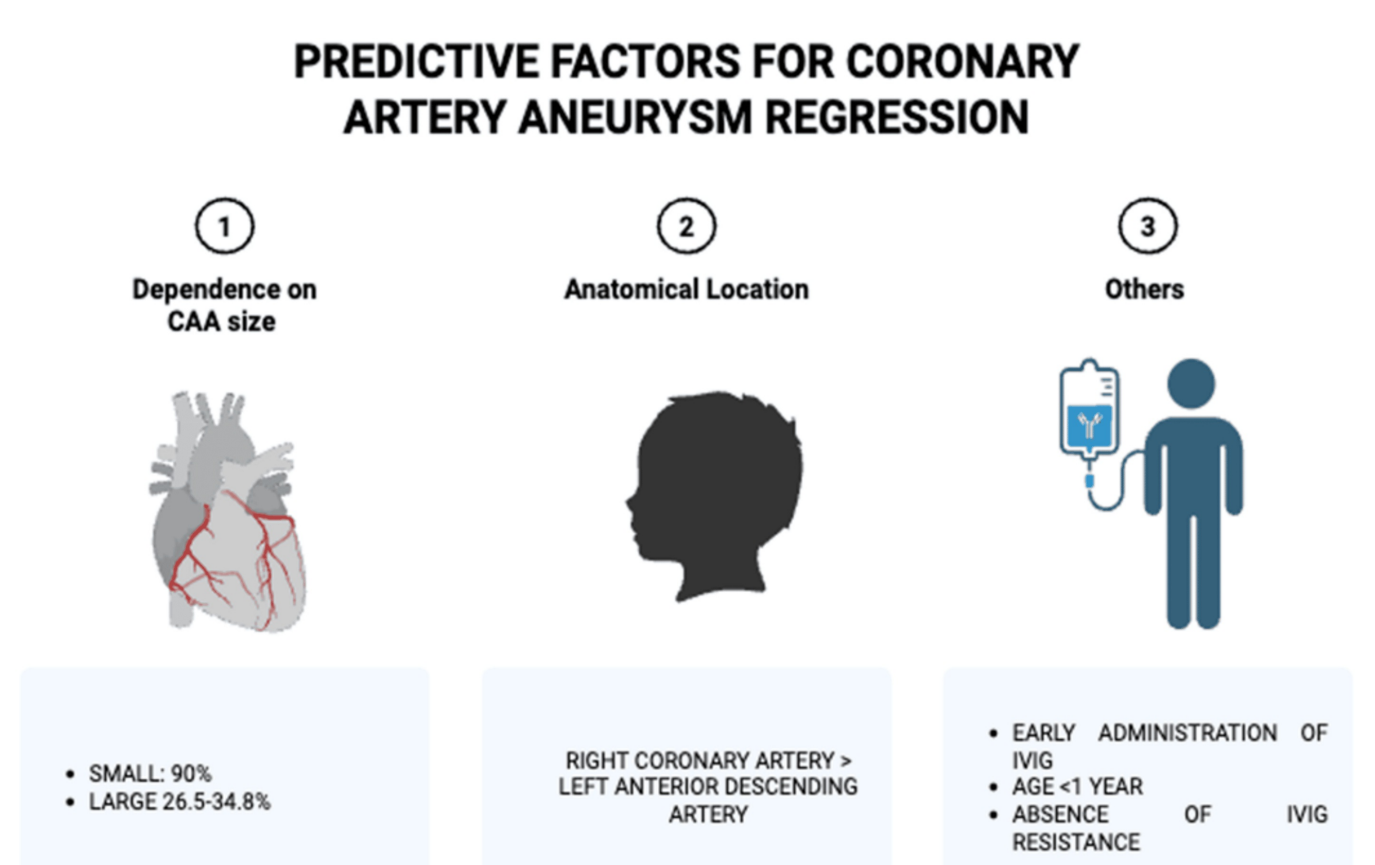
Small coronary aneurysms, aneurysms in the RCA, early administration of IVIG, and infants younger than one year are associated with higher rates of regression. Created with BioRender.com. RCA, right coronary artery; IVIG, intravenous immunoglobulin

**Figure 7:**
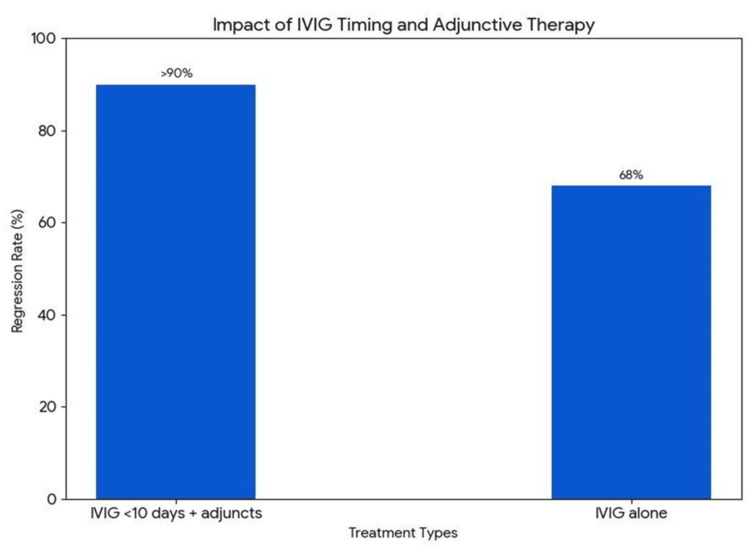
IVIG with adjuncts is associated with higher chances of regression (>90%) as compared to IVIG alone (68%). Original figure created by the authors. IVIG, intravenous immunoglobulin

CAA regression is a common outcome, but its rate is inversely proportional to the initial aneurysm size (Figure 6). While over 80% of small to moderate-sized aneurysms tend to regress, giant aneurysms rarely do(<35%). One study reported normalization rates of 99% for small, 92% for medium, and 57% for large CAAs over a 10-year follow-up [23]. Figure 7 shows that the use of adjunctive therapy is associated with higher rates of regression (~90%) as compared to IVIG alone (68%).

**Figure 8:**
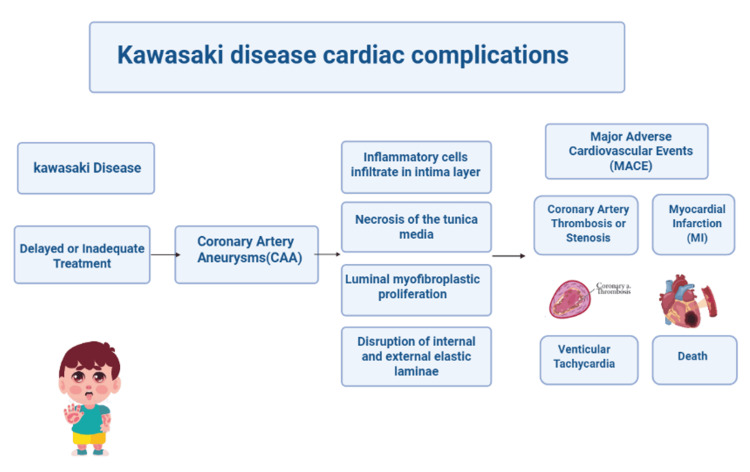
Delayed or inadequate treatment of KD is associated with higher chances of developing CAA with poor long term cardiovascular outcomes like MACE, Thrombosis, Stenosis, MI, Ventricular Tachycardia and Cardiac death. Created with BioRender.com. KD, Kawasaki disease; CAA, coronary artery aneurysm; MACE, major adverse cardiac event; MI, myocardial infarction

Importantly, luminal regression does not equate to normalization of the arterial wall, as layered thrombus and myofibroblastic proliferation can persist, leaving patients at risk for future cardiac events (Figure 8) [24]. The emergence of de novo CAAs (those appearing after initial IVIG treatment) may signify more severe arterial damage and prolong the time required for coronary artery normalization.

Long-Term Monitoring and Endothelial Health

Despite luminal regression, markers of endothelial injury persist for years after the acute KD episode. This persistence of elevated circulating endothelial cells (CECs) and endothelial microparticles (EMPs), even in patients whose CAAs have regressed or those who never developed CAAs, suggests an underlying state of subclinical vasculitis. This underscores the necessity for consistent and long-term cardiology follow-up for all KD survivors, irrespective of their initial CAA status. Notably, traditional cardiovascular risk factors, such as pulse-wave velocity and carotid intima media thickness, do not differ significantly from healthy controls in long-term KD survivors. Bilateral coronary artery involvement and complex aneurysm architecture (such as beaded CAAs) are significant independent risk factors for MACE [25].

Discussion

In this systematic review of 21 studies including 10,922 patients from seven countries (the United States, Canada, the United Kingdom, Japan, India, China, and Taiwan), we analyzed long-term cardiovascular outcomes in patients with CAA compared to those without aneurysms.

The incidence of CAA among patients with KD ranged between 3% and 27%, with a notably higher prevalence in infants less than 6 months (43.4%) and in males. Small and moderate aneurysms showed high regression rates (>80%), particularly within 5 years. Giant aneurysms, however, persisted in one third of the patients and were less likely to regress even after long-term follow-up.

Regression rates were slightly higher in the LAD (93.8%) compared to RCA (91.1%) for small aneurysms, but RCA showed higher regression for medium and large aneurysms. Notably, large aneurysms had poor regression in both arteries. Even in larger aneurysms, RCA regression rate (34.8%) was higher than LAD (26.5%), although it was not statistically significant.

Giant CAAs, determined by *Z*-score ≥10 according to the American Heart Association (AHA) or an absolute diameter ≥8 mm according to the Japanese Circulation Society (JCS) criteria, were consistently associated with worse outcomes, including thrombosis (45%), stenosis (16.7%), and major adverse cardiac events (MACE) (5%-14%) of patients.

The progression of CAA and occurrence of MACE depend on many factors, such as delayed IVIG therapy, IVIG resistance (10%-17%), larger baseline aneurysm diameter, and low albumin/globulin (A/G) ratio were linked with. On the other hand, a higher NLR showed good predictive power for CAL with 82% sensitivity and 80% specificity.

Administering IVIG within 10 days of illness, especially when combined with adjunctive therapies, leads to much higher aneurysm regression rates (>90%) compared to IVIG alone (68%). In a study, coronary artery bypass grafting (CABG) and percutaneous coronary intervention (PCI) were reported in 7.1% and 1.2% of the patients, respectively.

Randomized controlled trials demonstrated that the addition of corticosteroids to IVIG in high-risk groups significantly improved coronary *Z*-scores and reduced the incidence of CAA. Over the long term, cardiac event-free survival declined substantially in patients with giant aneurysms-from approximately 87.6% at one year to 50.7% at 10 years. Moreover, a significant proportion of patients with persistent CAAs experienced myocardial infarction (1.5%-16%), calcification (18%), or death from cardiovascular causes, emphasizing the importance of ongoing long-term surveillance.

Randomized controlled trials demonstrated significant improvements in coronary *Z*-scores and a reduced incidence of CAA associated with adding corticosteroids to IVIG in high-risk groups. Long-term outcomes: Cardiac event-free survival decreased over time in patients with giant aneurysms: (~87.6% at one year to 50.7% at 10 years). Also, a significant number of patients with persistent CAAs experienced Myocardial Infarction (1.5-16%), Calcification (18%), or death from cardiovascular causes, highlighting the critical need for long-term surveillance.

The results, as further explained below, demonstrate the associated risk of major adverse cardiovascular events (MACE), the prognostic value of aneurysm size, shape, and location, and the significance of early treatment with IVIG, to mention a few key points.

From the findings of these studies, the importance of long-term/ lifelong monitoring of patients with a history of KD cannot be overemphasized.

An examination of these studies revealed multiple risk factors associated with MACE. Aneurysm size, bilateral involvement, and a younger age at diagnosis were strongly correlated with adverse outcomes such as stenosis, thrombosis, and myocardial infarction [26-28]. The NLR and erythrocyte sedimentation rate (ESR) are valuable biomarkers for predicting the development of CAA as well as IVIG resistance. The results imply that routine inflammatory markers may assist in early risk stratification in clinical contexts.

Studies showed that CAA occurs in a wide range of patients with KD. This range reflects differences in treatment timing or patient populations. It was noted that younger children, especially those younger than 6 months, faced a higher risk of developing giant or medium CAAs [28,29]. This review highlights the vulnerability of patients with KD less than one year to CAAs. *Z*-score classification systems used globally demonstrate consistency in categorizing severity [30].

The regression of CAAs was noted to be dependent on the size of the CAA studies conducted, showing that small CAAs regressed for the most part, whereas large aneurysms often persisted. Aneurysms in the right coronary artery (RCA) regressed more often than those in the left anterior descending (LAD) artery, implying an anatomical influence on the outcome. Some predictive factors of regression include early administration of IVIG, age of one year or less, and the absence of IVIG resistance.

The foundation of treatment continues to be early and aggressive IVIG therapy. The incidence of CAA was significantly lower with a combination of IVIG and corticosteroids than with IVIG alone [13,23].

Alternative immunomodulatory therapies, including corticosteroids, anti-TNF alpha agents (e.g., infliximab, etanercept), and calcineurin inhibitors (e.g., ciclosporine), have been studied as treatment options for patients with IVIG-resistant disease [31]. However, the data on their long-term efficacy and safety remain limited and variable across different populations. Nevertheless, outcomes remain poorer when treatment is delayed or IVIG resistance is present [32].

The RCTs conducted endorsed the use of adjunctive corticosteroids to lower the incidence of CAA and Z-scores, especially among patients at high risk. Kobayashi’s research demonstrated a notable decrease in CAA formation with the use of prednisolone along with IVIG.

Patients with giant CAAs were the primary recipients of revascularization (CABG or PCI) procedures, with CABG being the most common intervention. Patients with large CAAs had a higher usage of antithrombotic agents (e.g., warfarin), which showed varying levels of success in event prevention [9,25].

Since its first description in 1967, KD has been the subject of extensive research. Despite various studies, considerable heterogeneity persists regarding its risk factors, pathophysiology, acute manifestations, and long-term cardiovascular sequelae. In our review, IVIG and aspirin continue to emerge as the cornerstone of initial management. IVIG effectively reduces the development of CAAs, while aspirin is employed for its antipyretic, antiplatelet, and anti-inflammatory properties. Although aspirin use in pediatric populations is generally limited due to concerns about Reye’s syndrome, its benefits in the context of KD are well-documented and outweigh the potential risks, supporting its continued inclusion in standard practice [7].

Echocardiography remains the preferred imaging modality for initial assessment and long-term follow-up due to its non-invasive nature and diagnostic utility [7,25]. It provides baseline and serial evaluations of coronary artery dimensions through *Z*-score and can help monitor for future complications such as thrombosis, aneurysm rupture, and myocardial ischemia [3]. Exercise stress echocardiography offers a safe tool for detecting ischemic changes during follow-up in high-risk populations [25]. Other imaging techniques, including CT angiography, cardiac MRI, and nuclear imaging, are available, although current evidence does not clearly favor this over echocardiography in routine practice.

CAA is the most severe cardiovascular complication of KD. The risk of adverse events increases with a greater aneurysm number (>3), complex morphology (e.g., beaded appearance), and larger size [6]. Giant aneurysms, defined as those with a *Z*-score of 10 or more, are associated with higher morbidity and mortality rates, and their development markedly worsens the overall prognosis [15]. Additionally, age appears to be an important susceptibility factor; infants diagnosed with KD are more prone to developing severe and giant CAAs compared to older children [10].

Diagnosis of KD remains clinical, as no single test definitively confirms the condition. Its clinical overlap with other febrile illnesses, including multisystem inflammatory syndrome in children (MIS-C), presents additional challenges to early recognition by physicians. Several inflammatory markers, such as NLR, C-reactive protein (CRP), and ESR, have been explored for their predictive value in identifying patients at risk for CAAs, though current evidence remains insufficient, and further large-scale studies are needed to validate their clinical utility [26,27,32].

Long-term management is critical in reducing KD-associated morbidity and mortality. Continued follow-up with pediatric cardiology and periodic imaging enables early detection of complications and guides therapeutic interventions. Although follow-up protocols vary, the need for monitoring strategies is clear, particularly in patients with persistent or giant aneurysms.

Despite significant advances, the pathophysiology of KD remains poorly understood. Emerging evidence has explored the involvement of molecular pathways, such as calcium-NFAT signaling, as well as immunologic and genetic contributors to disease development [28]. While promising, these findings require further validation through well-designed studies. Future advances in molecular and imaging technologies may offer critical insight into the mechanisms that lead to the development of this complex vasculitis.

The findings of this review are consistent with the current available literature and reinforce the importance of prompt diagnosis, timely treatment, and structured follow-up to mitigate long-term cardiovascular complications.

Communicable diseases were the leading cause of death a century ago. However, with advances in science, technology, and increased healthcare funding, cardiovascular diseases have become the leading cause of mortality in the 21st century in developed countries, affecting approximately 18 million people worldwide [33]. Although to a lesser extent, cardiovascular diseases also contribute to pediatric mortality, remaining a significant cause of death in infancy, particularly in cases of sudden cardiac death [15].

This highlights the importance and the relevance of conducting a systematic review that addresses the long-term cardiovascular outcomes in pediatric patients diagnosed with KD and the subsequent development of CAAs.

KD, named after the Japanese pediatrician Tomisaku Kawasaki, who first identified the association between recurring symptoms and its high incidence in the Japanese pediatric population, is an idiopathic syndrome that primarily affects children under the age of five. Although the acute phase is often self-limited, the disease can lead to serious cardiovascular complications with potential manifestations in adulthood. A rare but severe complication is the formation of CAAs, which affect approximately 0.3-0.5% of the population and may result in long-term cardiovascular morbidity and even mortality. Despite early diagnosis and prompt treatment with IVIG, a substantial number of affected children still develop coronary complications [23].

Current therapy is based on three main pillars. IVIG, administered as a single dose of 2 g/kg, typically leads to rapid clinical improvement within 24 hours. When given within the first 10 days of illness, IVIG significantly reduces the risk of coronary artery damage and prevents the formation of CAAs. The second key treatment is aspirin, initially given in high doses for its anti-inflammatory and antipyretic effects and subsequently continued at low doses for at least two months to prevent thrombus formation. Corticosteroids, once considered contraindicated due to concerns about increased risk of CAAs, are now recognized as beneficial in selected high-risk patients when used as adjunctive therapy. Recent studies, such as the RAISE TRIAL, have demonstrated that adding corticosteroids to standard IVIG treatment may reduce the incidence of CAAs and improve clinical outcomes in patients with predicted IVIG resistance.

The following review aims to explore the relationship between KD and the development of CAAs, and to evaluate the long-term cardiovascular outcomes of these patients. Drawing concrete conclusions from the current literature may help inform public health policies and guide the development of more effective pharmacological strategies, like reducing adverse drug effects, improving symptom control, and ultimately enhancing the quality of life for affected individuals. Moreover, it may provide valuable insights and evidence-based information for patients, families, and healthcare professionals.

Although numerous studies have investigated KD incidence, treatment modalities, and diagnostic strategies across different geographical regions, relatively few articles have focused on its long-term complications. By compiling and analyzing data from existing literature, this review seeks to highlight the progression and long-term cardiovascular effects of KD, including ischemic heart disease, myocardial infarction, sudden death, and related conditions. Furthermore, it underscores the need to establish standardized guidelines for effective treatment, follow-up duration, and monitoring strategies, whilst bridging existing knowledge gaps and promoting a more unified approach to disease management globally.

KD is a significant pediatric vasculitis with lifelong cardiovascular implications. Data from 24 global studies underscore the prevalence and progression of CAAs, their predictive markers, and the long-term outcomes in affected children. CAAs were observed in 3% to 25% of patients with KD, with giant aneurysms accounting for 3% to 8%. Notably, infants under 6 months of age demonstrated a disproportionately higher risk, up to 43% for developing medium to giant CAAs, emphasizing the need for age-specific clinical vigilance. Anatomical location [4] may also influence prognosis. Some studies suggest that aneurysms in the RCA tend to regress more frequently than those in the LAD artery, though further research is needed to confirm this trend. While more than 90% of small CAAs resolve over time, regression rates for large aneurysms remain lower, with estimates ranging between 26% and 35%.

Early administration of IVIG, ideally within the first 10 days of illness onset, is strongly associated with reduced aneurysm formation and higher rates of regression. Adjunctive corticosteroid therapy, as demonstrated in the RISE trial by Kobayashi et al., significantly lowered the incidence of CAAs (from 23% to 3%) and improved *Z*-scores in high-risk patients. Biomarkers such as the NLR and ESR have shown strong predictive value for CAA development and IVIG resistance, reinforcing their potential role in early risk stratification [34].

Long-term outcomes for patients with persistent large or giant CAAs are concerning. MACE-free survival rates at 30 years are estimated to range from 40% to 54%, with risks of myocardial infarction and mortality reaching 16% and 18%, respectively [35].

From a public health perspective, these findings emphasize the need for standardized protocols for KD screening, management, and follow-up. The establishment of KD registries and the implementation of *Z*-score-based risk stratification tools across healthcare systems would enhance consistency in care delivery. Ensuring equitable access to IVIG and corticosteroids, especially in resource-limited settings, should be prioritized. Furthermore, training frontline healthcare providers to recognize early clinical signs and initiate timely treatment is essential to reduce diagnostic delays and improve outcomes.

Future research efforts should focus on identifying genetic and molecular predictors of IVIG resistance and aneurysm progression. Large-scale, longitudinal studies following patients into adulthood are crucial for refining long-term surveillance strategies. Additionally, clinical trials evaluating novel biologic therapies, optimized anticoagulation regimens, and cost-effective monitoring protocols will be essential in improving long-term prognosis. In summary, the current body of evidence advocates for a comprehensive approach to KD, one that integrates early diagnosis, lifelong cardiovascular surveillance, and health policy reforms aimed at reducing the global burden of this vasculitis.

Limitations

This study has several limitations that warrant consideration. First, KD complicated by CAAs is relatively uncommon, and the small sample sizes of the included studies may limit the generalizability of findings due to reduced statistical power.

Second, the diagnostic modalities varied across studies. While echocardiography was the most used method due to its non-invasive nature and accessibility, its operator-dependent nature can lead to variability in image quality and interpretation. In contrast, computed tomography angiography (CTA) offers high-resolution imaging and superior anatomical detail but is less widely available and entails radiation exposure. These methodological differences likely introduced heterogeneity in diagnostic accuracy and may have influenced outcome precision.

Third, inconsistency in CAA classification criteria across studies represented another major limitation. Some investigations applied the Japanese Ministry of Health criteria, which are based on absolute coronary artery dimensions without adjusting for body surface area. Others followed the American Heart Association guidelines, which utilize *Z*-scores indexed to body surface area. These discrepancies may have contributed to inter-study variability in the reported incidence and severity of CAAs.

Additionally, variations in the dosage, timing, and type of IVIG therapy, considered the cornerstone of KD treatment, may have affected the outcomes. For instance, patients who received IVIG within the recommended 10-day window after symptom onset experienced fewer long-term cardiovascular complications. Furthermore, follow-up durations differed across studies, and some participants were lost to follow-up due to transition to adult care, a well-recognized challenge in the long-term management of chronic pediatric conditions. These factors may have led to inconsistencies in outcome reporting.

To address these limitations, future high-quality, randomized controlled trials with larger cohorts and extended follow-up periods are essential. Standardization of CAA classification criteria, the use of objective diagnostic tools, and consistency in treatment protocols are critical for enhancing comparability across studies. Moreover, stratifying data by age, gender, and baseline characteristics may help mitigate confounding and improve the validity of the findings. Such efforts are vital for the development of clear, evidence-based guidelines aimed at optimizing long-term outcomes and minimizing complications in children diagnosed with KD.

## Conclusions

KD is the leading cause of acquired heart disease in children in developed countries. Its etiology remains unclear, although genetic and infectious triggers are suspected. In patients with CAAs, major complications are largely confined to those with *Z*-scores >10. Infants under six months are at the highest risk. While small and medium CAAs often regress, large aneurysms carry a poorer prognosis. Early treatment with IVIG and aspirin significantly reduces CAA incidence. Adjunctive corticosteroids show promise in high-risk cases but require further study. Long-term surveillance and standardized care protocols remain critical to improving cardiovascular outcomes.

## Appendices

Appendix 

**Table 5:** PRISMA 2020 checklist. Source: [16]. This work is licensed under CC-BY 4.0. To view a copy of this license, visit https://creativecommons.org/licenses/by/4.0/

Section and Topic	Item #	Checklist item	Location where item is reported
TITLE			
Title	1	Identify the report as a systematic review.	1
ABSTRACT			
Abstract	2	See the PRISMA 2020 for Abstracts checklist.	3
INTRODUCTION			
Rationale	3	Describe the rationale for the review in the context of existing knowledge.	4-5
Objectives	4	Provide an explicit statement of the objective(s) or question(s) the review addresses.	5
METHODS			
Eligibility criteria	5	Specify the inclusion and exclusion criteria for the review and how studies were grouped for the syntheses.	6
Information sources	6	Specify all databases, registers, websites, organisations, reference lists and other sources searched or consulted to identify studies. Specify the date when each source was last searched or consulted.	6
Search strategy	7	Present the full search strategies for all databases, registers and websites, including any filters and limits used.	6
Selection process	8	Specify the methods used to decide whether a study met the inclusion criteria of the review, including how many reviewers screened each record and each report retrieved, whether they worked independently, and if applicable, details of automation tools used in the process.	6
Data collection process	9	Specify the methods used to collect data from reports, including how many reviewers collected data from each report, whether they worked independently, any processes for obtaining or confirming data from study investigators, and if applicable, details of automation tools used in the process.	6
Data items	10a	List and define all outcomes for which data were sought. Specify whether all results that were compatible with each outcome domain in each study were sought (e.g. for all measures, time points, analyses), and if not, the methods used to decide which results to collect.	8-9
	10b	List and define all other variables for which data were sought (e.g. participant and intervention characteristics, funding sources). Describe any assumptions made about any missing or unclear information.	NO
Study risk of bias assessment	11	Specify the methods used to assess risk of bias in the included studies, including details of the tool(s) used, how many reviewers assessed each study and whether they worked independently, and if applicable, details of automation tools used in the process.	6-8
Effect measures	12	Specify for each outcome the effect measure(s) (e.g. risk ratio, mean difference) used in the synthesis or presentation of results.	NO
Synthesis methods	13a	Describe the processes used to decide which studies were eligible for each synthesis (e.g. tabulating the study intervention characteristics and comparing against the planned groups for each synthesis (item #5)).	TABLE 1
	13b	Describe any methods required to prepare the data for presentation or synthesis, such as handling of missing summary statistics, or data conversions.	NO
	13c	Describe any methods used to tabulate or visually display results of individual studies and syntheses.	NO
	13d	Describe any methods used to synthesize results and provide a rationale for the choice(s). If meta-analysis was performed, describe the model(s), method(s) to identify the presence and extent of statistical heterogeneity, and software package(s) used.	NO
	13e	Describe any methods used to explore possible causes of heterogeneity among study results (e.g. subgroup analysis, meta-regression).	NO
	13f	Describe any sensitivity analyses conducted to assess robustness of the synthesized results.	NO
Reporting bias assessment	14	Describe any methods used to assess risk of bias due to missing results in a synthesis (arising from reporting biases).	7-8
Certainty assessment	15	Describe any methods used to assess certainty (or confidence) in the body of evidence for an outcome.	NO
RESULTS			
Study selection	16a	Describe the results of the search and selection process, from the number of records identified in the search to the number of studies included in the review, ideally using a flow diagram.	Figure 1
	16b	Cite studies that might appear to meet the inclusion criteria, but which were excluded, and explain why they were excluded.	NO
Study characteristics	17	Cite each included study and present its characteristics.	Table 1
Risk of bias in studies	18	Present assessments of risk of bias for each included study.	Figure-2, Figure-3 Table 3-5
Results of individual studies	19	For all outcomes, present, for each study: (a) summary statistics for each group (where appropriate) and (b) an effect estimate and its precision (e.g. confidence/credible interval), ideally using structured tables or plots.	NO
Results of syntheses	20a	For each synthesis, briefly summarise the characteristics and risk of bias among contributing studies.	NO
	20b	Present results of all statistical syntheses conducted. If meta-analysis was done, present for each the summary estimate and its precision (e.g. confidence/credible interval) and measures of statistical heterogeneity. If comparing groups, describe the direction of the effect.	8-9
	20c	Present results of all investigations of possible causes of heterogeneity among study results.	NO
	20d	Present results of all sensitivity analyses conducted to assess the robustness of the synthesized results.	NO
Reporting biases	21	Present assessments of risk of bias due to missing results (arising from reporting biases) for each synthesis assessed.	NO
Certainty of evidence	22	Present assessments of certainty (or confidence) in the body of evidence for each outcome assessed.	NO
DISCUSSION			
Discussion	23a	Provide a general interpretation of the results in the context of other evidence.	10-12
	23b	Discuss any limitations of the evidence included in the review.	15-16
	23c	Discuss any limitations of the review processes used.	15-16
	23d	Discuss implications of the results for practice, policy, and future research.	13-15
OTHER INFORMATION			
Registration and protocol	24a	Provide registration information for the review, including register name and registration number, or state that the review was not registered.	Not Registered
	24b	Indicate where the review protocol can be accessed, or state that a protocol was not prepared.	NA
	24c	Describe and explain any amendments to information provided at registration or in the protocol.	NA
Support	25	Describe sources of financial or non-financial support for the review, and the role of the funders or sponsors in the review.	NA
Competing interests	26	Declare any competing interests of review authors.	NA
Availability of data, code and other materials	27	Report which of the following are publicly available and where they can be found: template data collection forms; data extracted from included studies; data used for all analyses; analytic code; any other materials used in the review.	NO
